# Anti-Septics and Disinfectants

**Published:** 1881-06

**Authors:** 


### ANTI-SEPTICS AND DISINFECTANTS.

We are till now without any reliable disinfectant in the sense of being, at the same time, an anti-septic. The public generally confounds disinfectants—that is to say, compounds capable of destroying bad smells with substances efficacious to destroy morbid germs. In contagious maladies how frequently has chloride of lime been employed. It destroys a little some odors, but more especially marks them by its own, still its action on the germs of contagion is nil. Carbolic acid should be employed in preference, but even this is not efficacious as an anti-putrescent unless diffused in such quantities in an atmosphere as to render respiration impossible, being dangerous. And following Sternbery's experiments it is not certain if carbolic acid can kill all species of disease germs. Ozone and other oxidants are excellent, but their value depends on their being employed in high doses; and if so employed they grip the throat and irritate the tissues. Mr. Peyrusson claims to have discovered a product certain at once as disinfectant and an anti-septic, while producing no irritation of the tissues: one-eighth of an ounce is sufficient to disinfect an apartment combining 100 yards. His substance is azotized ether, being the product of a mixture of azotic acid of 36 deg. and four parts of alcohol at 90 deg. Mr. Peyrusson has placed in vases beaten eggs, blood, meat, etc., and allowed putresence to do its work. He next placed in the vases a bottle, unstoppered, containing azotized ether, and they protected the contents intact from further decay, while a vase left free advanced to the last stages of decomposition. The experiments were renewed with chloride of lime, carbolic acid, and azone: the first did not stop putrefaction, the second delayed it, the third checked decay at first, but after three days it was inefficacious. The azotized ether proved faultless; but it has also been tried in some hospital wards, and with marked success. At Limoges, in an hospital ward cubing 300 yards, and containing 12 beds, the atmosphere was positively repulsive: 3 ounces of the ether poured out on as many saucers completely purified the air, and hastened the recovery of the inmates. Similar results have taken place in other hospitals.

